# Physiological Response of *Crocosphaera watsonii* to Enhanced and Fluctuating Carbon Dioxide Conditions

**DOI:** 10.1371/journal.pone.0110660

**Published:** 2014-10-24

**Authors:** Mary R. Gradoville, Angelicque E. White, Ricardo M. Letelier

**Affiliations:** Oregon State University, College of Earth, Ocean, and Atmospheric Sciences, Corvallis, Oregon, United States of America; Loyola University Medical Center, United States of America

## Abstract

We investigated the effects of elevated *p*CO_2_ on cultures of the unicellular N_2_-fixing cyanobacterium *Crocosphaera watsonii* WH8501. Using CO_2_-enriched air, cultures grown in batch mode under high light intensity were exposed to initial conditions approximating current atmospheric CO_2_ concentrations (∼400 ppm) as well as CO_2_ levels corresponding to low- and high-end predictions for the year 2100 (∼750 and 1000 ppm). Following acclimation to CO_2_ levels, the concentrations of particulate carbon (PC), particulate nitrogen (PN), and cells were measured over the diurnal cycle for a six-day period spanning exponential and early stationary growth phases. High rates of photosynthesis and respiration resulted in biologically induced *p*CO_2_ fluctuations in all treatments. Despite this observed *p*CO_2_ variability, and consistent with previous experiments conducted under stable *p*CO_2_ conditions, we observed that elevated mean *p*CO_2_ enhanced rates of PC production, PN production, and growth. During exponential growth phase, rates of PC and PN production increased by ∼1.2- and ∼1.5-fold in the mid- and high-CO_2_ treatments, respectively, when compared to the low-CO_2_ treatment. Elevated *p*CO_2_ also enhanced PC and PN production rates during early stationary growth phase. In all treatments, PC and PN cellular content displayed a strong diurnal rhythm, with particulate C:N molar ratios reaching a high of 22∶1 in the light and a low of 5.5∶1 in the dark. The *p*CO_2_ enhancement of metabolic rates persisted despite *p*CO_2_ variability, suggesting a consistent positive response of *Crocosphaera* to elevated and fluctuating *p*CO_2_ conditions.

## Introduction

Anthropogenic emissions and land use change are increasing the concentration of carbon dioxide (CO_2_) in the atmosphere and surface ocean waters [1]. The predicted effects of elevated CO_2_ partial pressure (*p*CO_2_) and consequent ocean acidification (OA) on marine ecosystems include a potential upregulation of metabolic processes by CO_2_-limited phytoplankton species [2]. Because the carboxylating enzyme ribulose-1,5-bisphosphate carboxylase-oxygenase (RuBisCO) is typically not saturated under ambient surface seawater *p*CO_2_, many phytoplankton groups invest energy in carbon concentrating mechanisms (CCMs) to increase CO_2_ concentrations at the catalytic site [3]. Hence, under future OA conditions, phytoplankton with low-affinity RuBisCO could potentially down-regulate CCMs and reallocate energy and elemental resources to allow for increased carbon (C) fixation and growth rates [4]. Indeed, elevated *p*CO_2_ has been shown to stimulate C fixation by select monocultures of phytoplankton and natural assemblages (reviewed in [5]).

Elevated *p*CO_2_ appears to have a particularly strong metabolic enhancement in dinitrogen (N_2_)-fixing (diazotrophic) cyanobacteria. Initial laboratory experiments using strain IMS101 of *Trichodesmium*, a group of filamentous, non-heterocystous cyanobacteria, showed that doubling *p*CO_2_ increases N_2_ fixation rates by 35–138% and C fixation rates by 23–40% [4], [6]–[8]. Likewise, the unicellular cyanobacterium *Crocosphaera* strain WH8501 displays increased rates of C and N_2_ fixation under elevated *p*CO_2_ conditions [9]. *Trichodesmium* and *Crocosphaera* are dominant diazotrophic taxa in oligotrophic open-ocean environments [10], where the bioavailable nitrogen (N) from N_2_ fixation can fuel up to half of production exported from the euphotic zone [11]. In theory, a global *p*CO_2_ enhancement of marine N_2_ fixation could increase oceanic C uptake and export, producing a negative feedback to climate change [6].

In contrast to laboratory findings, recent field experiments using natural diazotrophic assemblages do not reliably show that raising *p*CO_2_ enhances N- or C-based productivity. Elevating *p*CO_2_ in bottle incubations has been shown to increase N_2_ fixation rates by *Trichodesmium* colonies isolated from the Subtropical Atlantic and the Gulf of Mexico [12], [13] but not by *Trichodesmium* colonies isolated from the North Pacific Subtropical Gyre [14]. Furthermore, experiments using whole water diazotrophic assemblages from the North and South Pacific gyres have found no relationship between *p*CO_2_ and N_2_ fixation rates [15], [16]. The inconsistent results from field incubations highlight the importance of assessing how the effect of *p*CO_2_ on diazotrophs is influenced by other factors, including community composition [14], [17], physiology, and environmental conditions [18].

One methodological challenge in *p*CO_2_ manipulation studies is producing realistic timescales for *p*CO_2_ perturbations. In nature, seawater *p*CO_2_ varies spatially and temporally: phytoplankton experience *p*CO_2_ fluctuations on diurnal, seasonal, episodic, and long-term (e.g. OA-driven) timescales [19]. However, most laboratory OA studies grow cultures under stable *p*CO_2_ treatments, allowing cultures to acclimate to a steady *p*CO_2_ for multiple generations. These stable *p*CO_2_ conditions may affect phytoplankton differently than the dynamic *p*CO_2_ experienced in marine ecosystems; for instance, energetic costs associated with resource allocation may be minimized under stable *p*CO_2_ regimes [20].

Predicting the future response of diazotrophic assemblages to OA will require an assessment of how *p*CO_2_ affects marine diazotrophs under variable environmental conditions and physiological states. Here, we present data from experiments tracking the growth, PC and PN production rates of *Crocosphaera watsonii* WH8501 cultures bubbled with air at three CO_2_ levels (∼400, 750,1000 ppm), while allowing for biologically induced *p*CO_2_ variability in the culture medium resulting from photosynthesis and respiration. This approach contributes to the existing literature on potential responses of marine diazotrophs to future OA, but expands from previous studies by testing the effect of elevated *p*CO_2_ under a dynamic *p*CO_2_ environment. Our results suggest a consistent response of this organism to elevated *p*CO_2_ under variable *p*CO_2_ conditions.

## Methods

### Culture conditions

Unialgal stock cultures of *Crocosphaera watsonii* strain WH8501 were grown in 0.2 *µ*m-filtered, nitrogen-free YBCII medium [21] using 40 *µ*mol L^−1^ K_2_HPO_4_. Cultures were not axenic, but heterotrophic bacterial counts were kept at low levels (1.4–2.1×10^5^ cells mL^−1^). Light was provided using cool white fluorescent bulbs set on a 12∶12 light/dark cycle. Stock cultures were grown at 24°C and 250 *µ*mol quanta m^−2^ s^−1^. For the experiment, cultures were grown at 30°C, a temperature which promotes optimal growth of *Crocosphaera* in the laboratory [22] and at which high abundances of *Crocosphaera* cells have been observed at sea [23]. Incoming irradiance for the experiment was 1000 *µ*mol quanta m^−2^ s^−1^ as measured by a Biospherical light meter, a level which is saturating but not inhibitory for *Crocosphaera* WH8501 [24]. Cultures were stirred at least once a day with magnetic stir bars to minimize cells sticking to the glass. The *p*CO_2_ was manipulated by gently bubbling cultures with commercially prepared air/CO_2_ mixtures of ∼400 ppm (‘low-CO_2_’), ∼750 ppm (‘mid*-*CO_2_’), and ∼1000 ppm (‘high-CO_2_’). Parent cultures were grown under these CO_2_, light, and temperature conditions for seven days (∼3–4 generations) before productivity rates were measured.

### Experimental Design

*Crocosphaera* cultures were grown under three CO_2_ treatments and monitored over a six-day period (day 0–day 5). Triplicate bottle replicates were used for each CO_2_ treatment. Preceding the experiment, 2 L glass bottles were filled with 0.2 *µ*m-filtered media that was pre-equilibrated to target *p*CO_2_ levels. Initial CO_2_ equilibration of the media was verified by measuring a stable *p*CO_2_ in outflowing gas (>24 h equilibration) using a LI-840 LI-COR gas analyzer (Biosciences). The pH of each replicate was measured and converted through CO2calc (see below) to produce initial *p*CO_2_ values of 404±23, 724±51, and 916±34 *µ*atm for low-, mid-, and high-CO_2_ treatments, respectively. To initiate the experiment, parent cultures in exponential growth phase were diluted into the pre-equilibrated media, producing initial biomass concentrations of 3.9–4.6 *µ*g chlorophyll *a* (Chl *a*) L^−1^ (Table 1). Because parent cultures were shifting media *p*CO_2_ through biological processes, the addition of parent cultures to pre-equilibrated media altered the initial *p*CO_2_ values to 355±14, 600±21, and 788±11 *µ*atm (Table S1). Bottles were gently bubbled with air/CO_2_ mixtures at 50 mL min^−1^ throughout the experiment. Each replicate was sampled once daily after the sixth hour of light (L6) from day 0–day 2, then four times daily after the sixth and twelfth hours of light and darkness (L6, L12, D6, and D12) from day 3–day 5. The pH in each replicate was measured at the time of sampling and samples were preserved for particulate carbon (PC), particulate nitrogen (PN), Chl *a*, and flow cytometric cell counts (FCM).

**Table 1:** Time series biomass measurements for cultures of *Crocosphaera watsonii* WH8501 grown under three CO_2_ treatments.

	Low-CO_2_ treatment				Mid-CO_2_ treatment				High-CO_2_ treatment			
Time point	PC	PN	Cells	Chl *a*	PC	PN	Cells	Chl *a*	PC	PN	Cells	Chl *a*
Day 0 L6	124 (12)	8.8 (1)	-	4.3 (0.6)	120 (4)	8.5 (0.7)	-	4.6 (0.3)	118 (2)	7.6 (0.1)	-	3.9 (0.4)
Day 1 L6	166 (24)	14.2 (2)	5.6E+05 (7.4E+04)	7.3 (1.6)	192 (1)	16.2 (0.2)	8.0E+05 (6.3E+04)	9 (0.4)	172 (21)	13.2 (2)	7.5E+05 (1.9E+04)	7.9 (0.4)
Day 2 L6	374 (145)	22 (4)	7.4E+05 (1.2E+05)	11.6 (1.5)	424 (73)	28.2 (3)	1.2E+06 (1.1E+05)	16.3 (3)	547 (63)	28.3 (2)	1.2E+06 (9.7E+03)	15.1 (1)
Day 3 L6	542 (65)	42.5 (6)	1.4E+06 (2.5 E+05)	24.8 (3)	706 (88)	55.4 (6)	2.4E+06 (1.9E+05)	39.6 (6)	740 (44)	55.9 (4)	2.6E+06 (1.3E+05)	35.3 (6)
Day 4 L6	833 (121)	66 (7)	2.3E+06 (3.1E+05)	31.6 (2.6)	1144 (174)	91.7 (10)	3.6E+06 (1.5E+06)	50.6 (9)	1308 (121)	97.4 (7)	4.8E+06 (2.1E+05)	49.1 (9)
Day 5 L6	1037 (58)	84.2 (3)	3.3E+06 (2.9E+05)	55.4 (2.1)	1589 (186)	130.2 (12)	6.7E+06 (4.4E+05)	82.5 (8)	1852 (65)	155.1 (2)	8.1E+06 (3.8E+05)	101 (2)

Concentrations of particulate carbon (*µ*mol L^−1^; PC), particulate nitrogen (*µ*mol L^−1^; PN), cells (# mL^−1^), and chlorophyll *a* (Chl *a*; *µ*g L^−1^) are provided for L6 time points. Data are mean values from three replicate bottles; standard deviations are presented in parentheses.

### Analytical Measurements

For PC/PN and Chl *a* measurements, three subsamples of 5–50 mL (depending on cell density) were withdrawn from each replicate and filtered onto glass fiber filters (GF/F, Whatman), using pre-combusted GF/F filters for PC/PN. Samples were immediately frozen at −80°C (PC/PN) or −20°C (Chl *a*). PC/PN samples were dried at 60°C overnight, packaged into silver and tin capsules, and analyzed using a Carlo Erba elemental analyzer. Acetanilide (71.09% C and 10.36% N by weight) served as a standard, and filter blanks were <10% of total C and N content. Chl *a* was extracted in 90% acetone at −20°C for 48 hours and analyzed with a Turner Model 10-AU fluorometer using the acidification method of Strickland and Parsons [25]. On day 0 and day 5 L6 time points, 25 mL samples were withdrawn from GF/F filtrate and immediately frozen for soluble reactive phosphorus (assumed to be equivalent to PO_4_) and NH_4_ analyses. NH_4_ concentrations were measured with a Technicon AutoAnalyzer II, using a modified indophenol blue method [26] and PO_4_ via the standard ascorbic acid-molybdate method [25].

*Crocosphaera* and heterotrophic bacterial cell densities were measured using FCM. Two 3-mL subsamples were withdrawn from each replicate, pipetted into 4 mL cryovials, and fixed with paraformaldehyde at a final concentration of 1% (volume volume^−1^). Samples were inverted and allowed to sit in the dark for ∼10 minutes before being frozen at −80°C. For analysis of *Crocosphaera* cell densities, samples were thawed on ice in the dark then spiked with a known number of 3 *µ*m Polysciences Fluoresbrite yellow-green beads and run on a Becton-Dickinson FASCaliber flow cytometer with a 488 nm laser. *Crocosphaera* cells and beads were distinguished from other particulate matter by their side light scatter and fluorescence in orange wavelengths. The bead count determined the volume of sample run, and thus the concentration of *Crocosphaera* cells. A similar method was used to enumerate the background heterotrophic bacteria in these cultures. The samples were spiked with Fluoresbrite 1 *µ*m beads, stained with SYBR Green I according to the method of Marie et al. [27], and differentiated by their side light scatter and green fluorescence.

The pH of each replicate was measured directly using a VWR sympHony electrode calibrated with VWR buffers (NBS scale). pH values were converted to *p*CO_2_ by assuming a constant total alkalinity (TA) for the YBCII medium (2500 *µ*M). This TA value was determined by analyzing DIC and *p*CO_2_ of a separate batch of YBCII medium according to the methods of Bandstra et al. [28], then the program CO2calc [29] was used to convert DIC and *p*CO_2_ to TA (with CO_2_ constants from Merbach et al. [30] refit by Dickson and Millero [31], and a correction to the NBS scale by the CO2calc program). Finally, CO2calc was used to calculate *p*CO_2_ from our measured pH data and the constant TA.

Our assumption of constant TA of the YBCII medium throughout the experiment is based on the observation that bubbling with air/CO_2_ mixtures perturbs DIC but not TA [32]; thus, any change in TA through the experiment was due to biological activity. While the process of N_2_ fixation does not affect TA, photosynthesis can have a small effect due to hydrogen ion uptake to balance anionic nutrient (N, P, and S) acquisition [33]. Assuming no inorganic N uptake (as cultures were grown in N-free media) and a 2.4∶1 S:P uptake ratio (as in [33]), we estimate that the average PO_4_ drawdown of ∼9 *µ*M observed in our experiment (see *Results and Discussion*) increased TA by an average of ∼52* µ*M by day 5, generating a maximum *p*CO_2_ error of ∼2%. In addition, we assume that calcium carbonate (CaCO_3_) minerals were not precipitated in our experiment, as the presence of PO_4_ has been shown to inhibit CaCO_3_ precipitation, even at high CaCO_3_ saturation states [34], [35].

### Rate calculations

**Figure 1:**
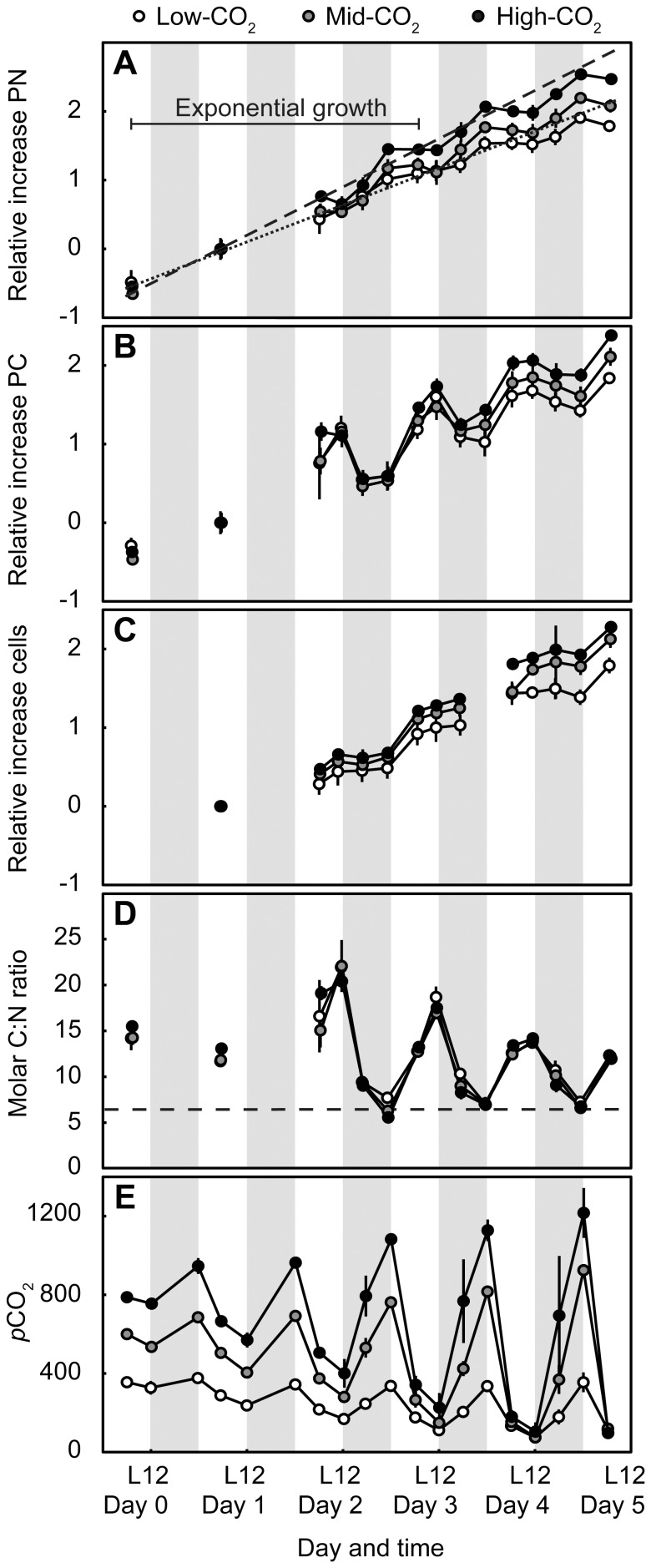
Growth of *C. watsonii* WH8501 batch cultures over a 6-day period under three CO_2_ treatments. Shown are concentrations of PN (a), PC (b), and cells (c), molar C:N ratios (d), and *p*CO_2_ in *µ*atm (e) within each treatment. For (a–c), the concentrations for each time point (Table S1) were first normalized to the concentration at the day 1 L6 time point, then ln-transformed. The derived slopes between day 1 L6 and day 3 L6 time points correspond to the exponential growth rates (*µ*) as shown in Table 2. The lines in (a) represent linear regressions through the day 1, day 2, and day 3 L6 time points for high-CO_2_ (dashed line) and low-CO_2_ (dotted line) treatments. The regression lines have been extended to the full time period (day 0–5) for visualization of exponential growth (day 0–3 L6 time points) transitioning to early stationary growth (L6 time points after day 3). The dotted line in (d) represents the 6.6 C:N ratio expected from Redfield stoichiometry. Shaded areas represent the dark periods. Error bars represent standard deviations from three replicates.

Specific growth rates were calculated for each of the biomass parameters measured: cell density, PC, PN, and Chl *a* (Table 2). Growth rates (*μ*) were determined using Eq. 1,
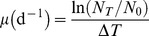
(1)where *N_T_* is the biomass at day 3, *N_0_* is the biomass at day 1, and Δ*T* is the time interval in days. The day 1–day 3 time interval was chosen for growth rate calculations because this was the phase of exponential growth (Fig. 1A).

**Table 2:** Biomass-specific growth rates of *Crocosphaera watsonii* WH8501 cultures grown under three CO_2_ treatments.

	Specific Growth rate *µ* (d^−1^)			Tukey HSD *p*-value	
	Low-CO_2_	Mid-CO_2_	High-CO_2_	Low-CO_2_ vs. high-CO_2_	Low-CO_2_ vs. mid-CO_2_
Cell density	0.45 (0.02)	0.54 (0.02)	0.60 (0.02)	<0.001	<0.01
Particulate nitrogen	0.54 (0.02)	0.60 (0.05)	0.71 (0.04)	<0.01	0.2
Particulate carbon	0.58 (0.02)	0.63 (0.06)	0.71 (0.06)	<0.05	0.44
Chlorophyll *a*	0.60 (0.06)	0.72 (0.05)	0.72 (0.07)	0.12	0.14

Rates were calculated from L6 time points between day 1 and day 3 (exponential growth phase). Tukey HSD *p*-values are provided for comparisons among CO_2_ treatments. Standard deviations of growth rates from three replicate bottles are presented in parentheses.

Carbon-normalized PC and PN production rates (∼net C and N_2_ fixation rates) were calculated using Eq. 2,
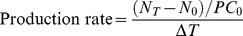
(2)where *N_T_* is the biomass (PC or PN) at the final time point, *N_0_* is the biomass at the initial time point, *PC_0_* is the initial PC concentration, and Δ*T* is the time interval in days. Production rates were calculated for both exponential (day 1–day 3) and early stationary (day 3–day 5) growth phases.

Growth rates, PC and PN production rates were all calculated using data from L6 time points. Day 0 was excluded from these analyses due to missing FCM samples on this day.

### Statistics

The effects of *p*CO_2_ on growth rates, PC production, PN production, and molar C:N ratios were assessed using the one-way ANOVA. Differences between CO_2_ treatments were determined using the Tukey Honest Significance Difference (HSD) test of multiple comparisons. All data reported in this study are averages from triplicate bottles. Statistical tests were run using the program R (http://www.r-project.org/).

## Results and Discussion

**Figure 2:**
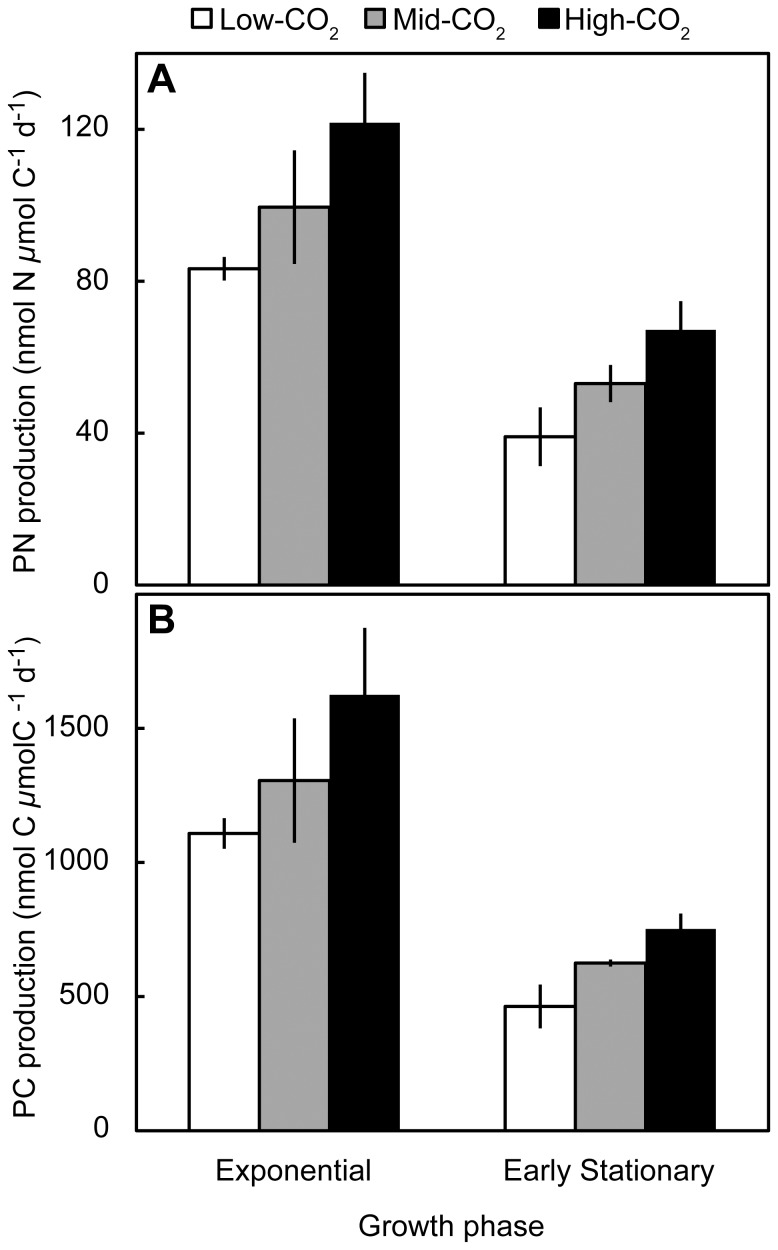
Carbon-normalized PN (a) and PC production rates (b) of *Crocosphaera* WH8501 cultures grown under three CO_2_ treatments during periods of exponential (day 1–day 3) and early stationary (day 3–day 5) growth phases. Production rates are calculated as increases in PC and PN concentrations (data provided in Table 1) per time normalized to initial PC concentrations within the time interval. Error bars represent standard deviations from three replicates.

Our study tested how enhanced *p*CO_2_ affects the growth, PC and PN production rates of high-density *Crocosphaera* cultures. In agreement with a previous study [9], we found that PC production, PN production, and growth rates were all positively correlated with *p*CO_2_ (Table 2, Fig. 2). This *p*CO_2_ enhancement was observed for *Crocosphaera* cultures during both exponential and early stationary growth phases (Fig. 2). The high growth rates and cell densities observed in our study produced a strong diurnal rhythm of C and N metabolism in *Crocosphaera* cultures as well as daily *p*CO_2_ variability (Fig. 1).

### Diurnal rhythm in growth and *p*CO_2_

Diazotrophic cyanobacteria employ various mechanisms to separate the oxygen (O_2_) evolved through photosynthesis from the enzyme nitrogenase, which catalyzes biological N_2_ fixation and is irreversibly inactivated by O_2_ [36]. *Crocosphaera* circumvents this problem by restricting N_2_ fixation to the nighttime, when O_2_ is not being produced. The energy needed to fix N_2_ is generated photosynthetically in the light and stored primarily as carbohydrate granules [37]; respiration of these organic C reserves fuels N_2_ fixation in the dark. The temporal separation and energetic linkage of photosynthesis and N_2_ fixation in *Crocosphaera* produces a daily pattern in the timing and magnitude of PC and PN production and loss. In our study, cultures were grown under high light conditions, producing high growth rates and an especially pronounced diurnal rhythm.

We observed a strong daily pattern in PC production, PN production, and cell division by *Crocosphaera* in all CO_2_ treatments (Fig. 1). The PC concentration of *Crocosphaera* cultures fluctuated widely between the light and dark periods: PC increased 48–216% in the light (∼C fixation) and decreased 17–79% in the dark (∼respiration) (Fig. 1B). This substantial dark PC loss is consistent with previous studies of *Crocosphaera* and reflects the respiration of carbohydrate reserves to fuel N_2_ fixation [37]–[39]. A fraction of PC may have also been exuded from cells, as it has been shown that *Crocosphaera* WH8501 can release ∼10% of total C content daily as extracellular polymeric substances [38]; however, dissolved organic C was not measured in our study. The sharp increase in cell concentration following the D12 measurement indicates that cells divided in the first half of the light period (Fig. 1C).

PN production was restricted to the dark period, when PN concentrations increased between 48 and 93% (Fig. 1A). Rates of PN increase approximate net N_2_ fixation rates, though we cannot rule out NO_3_ or NH_4_ utilization as driving some small fraction of PN production. While cultures were grown in N-free media [21], the initial dilution of the parent cultures into fresh media resulted in NH_4_ concentrations of 0.3±0.1 *µ*M on day 0. By day 5, NH_4_ had increased to 1.1±0.3 *µ*M, supporting the interpretation that the rate of accumulation of inorganic plus particulate N in our cultures was fueled by N_2_ fixation. The accumulated NH_4_ had presumably been fixed by *Crocosphaera* and released from cells; *Crocosphaera* have been previously observed to release 23–67% of recently fixed N [38]. PN decreased slightly (1–8%) during the light period of the experiment (Fig 1A), which may indicate daily NH_4_ release. The total NH_4_ accumulated through the experiment (0.8 *µ*M) represents 0.5–1% of total PN production (day 5–day 0, Table 1).

Together, the high rates of PN production, PC production in the day (∼C fixation) and PC loss in the night (∼C respiration) led to large fluctuations in the particulate C:N ratio over the daily cycle: molar C:N ratios in our study ranged from 5.5–22.1 (Fig. 1D). The C:N ratios at D12 time points had a relatively consistent range (∼5.5–8) encompassing the 6.6 ratio predicted from Redfield stoichiometry [40]. Elevated C:N ratios at L6, L12, and D6 time points were driven by the accumulation of organic C reserves to fuel dark N_2_ fixation and other cellular processes. These daily C:N deviations were independent of *p*CO_2_ treatment. Previous studies have reported less dramatic stoichiometric fluctuations in *Crocosphaera*, with daily C:N content ranging from 6.5–8.5 [39] and 5.0–8.8 [38]. The strong daily C:N deviations observed in our study are consistent with metabolic rates at high growth rates (∼0.5 d^−1^) of cultures grown at optimum temperature (30°C, [20]) and saturating incoming irradiance (1000 *µ*mol quanta m^−2^ s^−1^, [24]). Our experiment spanned both exponential and early stationary growth phases. Cultures grew exponentially from day 0 to day 3, at which point growth rates began to decline (Fig. 1A). The shift in growth phase is evident from non-linearity of natural log-normalized PN growth curves at L6 time points (Fig. 1A) and from decreased PC and PN production rates from day 3–day 5 (Fig. 2). Declines in the magnitude of daily PC fluctuations indicate decreased rates of photosynthesis (∼positive derivative, in light) and respiration (∼negative derivative, in dark) after day 3 (Fig. 1B). *Crocosphaera* cultures were grown in an artificial medium initially containing an ample supply of macro and micronutrients [16], and PO_4_ remained replete throughout the experiment (PO_4_ decreased from 36±1.8 *µ*M on day 0 to 27±1.8 *µ*M on day 5, data not shown). Thus, although we cannot exclude the possibility of limitation by a micronutrient, we hypothesize that the shift to early stationary growth phase resulted from self-shading or a decreased RuBisCO carboxylation efficiency during the second half of the photoperiod, either through direct CO_2_ limitation or through competitive inhibition of carboxylation from photorespiration at high O_2_:CO_2_ ratios [41].

The high *Crocosphaera* growth rates and cell densities affected the stability of C chemistry within CO_2_ treatments: photosynthesis and respiration produced *p*CO_2_ variability despite continuously bubbling cultures with air/CO_2_ mixtures (Fig. 1E). The magnitude of *p*CO_2_ variability increased throughout the experiment, and by day 5, the high-CO_2_ treatment had fluctuated between 1216 and 96 *µ*atm (Fig. 1E). In addition, by day 4, all cultures, independent of *p*CO_2_ treatment, reached consistent minimum *p*CO_2_ values close to 100 *µ*atm in measurements taken at the end of the light cycle (L12); hence, ∼100 *µ*atm appears to represent a physiological limit for the uptake of inorganic C by this organism. Despite the extreme *p*CO_2_ fluctuations within treatments, mid- and high-CO_2_ treatments always had higher *p*CO_2_ values than the low-CO_2_ treatment (which fluctuated between 376 and 74 *µ*atm), with the exception of the final time point. To separate the effect of inflowing *p*CO_2_ from the possible confounding effect of cell densities, we present growth rates from day 1 to day 3 when *p*CO_2_ fluctuations were less extreme and treatments did not overlap in *p*CO_2_ range (Table 2); the mean *p*CO_2_ levels measured in this time interval were 252, 477, and 665 *µ*atm for the low-, mid-, and high-CO_2_ treatments, respectively. The response of *Crocosphaera* to elevated *p*CO_2_ combined with daily, biologically induced *p*CO_2_ fluctuations may have ecological implications for bloom scenarios (discussed below).

### *p*CO_2_ enhancement of growth, PC and PN production

Consistent with previous studies of *Crocosphaera* WH8501 [7] and *Trichodesmium* IMS101 [4], [6]–[8], we found that elevating *p*CO_2_ increased *Crocosphaera* growth, PC and PN production rates (Table 2, Fig. 2). Growth in the high-CO_2_ treatment was significantly higher than in the low-CO_2_ treatment for growth rates specific to PC, PN, and cell density (Table 2). Cell-specific growth rates were also significantly higher in the mid-CO_2_ treatment than the low-CO_2_ treatment (Table 2). Chl *a* growth rates were not significantly different between CO_2_ treatments, possibly because of the large coefficient of variation between replicates (7–10%) (Table 2). Biomass-normalized PC and PN production rates were significantly enhanced in mid-CO_2_ and high-CO_2_ treatments compared to the low-CO_2_ treatment (Fig. 2). The *p*CO_2_ enhancement of PC and PN production rates was observed both during exponential (day 1–day 3) and early stationary (day 3–day 5) growth phases (Fig. 2).

The only previous CO_2_ manipulation study using *Crocosphaera* strain WH8501 tested a *p*CO_2_ range of 190–50 *µ*atm and observed that raising ambient *p*CO_2_ (380 *µ*atm) to 750 *µ*atm produced 1.2- and 1.4-fold higher rates of C and N_2_ fixation, respectively [9]. In our study, PC and PN production rates during exponential growth were both ∼1.2-fold higher in the mid-CO_2_ treatment than the low-CO_2_ (Fig. 2). The magnitude of *p*CO_2_ enhancement we observed is strikingly similar to those reported by Fu et al. [9], especially considering that the environmental conditions utilized in our study differed from the low light (80 *µ*mol quanta m^−2^ s^−1^), steady *p*CO_2_ conditions of Fu et al. [9]. In our study, including a higher *p*CO_2_ treatment displayed even larger enhancements: both PC and PN production rates were ∼1.5-fold higher in the high-CO_2_ treatment than the low-CO_2_ treatment. Higher *p*CO_2_ treatments would need to be included to determine the threshold *p*CO_2_ condition that saturates C and N_2_ fixation rates of *Crocosphaera* WH8501.

### Conclusions and ecological implications

We observed that elevated *p*CO_2_ conditions significantly enhanced PC production, PN production, and growth rates of *Crocosphaera* strain WH8501. This *p*CO_2_ enhancement persisted despite biologically induced *p*CO_2_ variability in all treatments. By allowing photosynthesis and respiration to drive *p*CO_2_ deviations from target values, our methods contrast with those of many previous OA studies, which often keep cultures optically dilute and/or do not report the measured *p*CO_2_ time course for each replicate. Though *p*CO_2_ in our study varied within treatments, the mid- and high-CO_2_ treatments had higher *p*CO_2_ values than the low-CO_2_ treatment for nearly all time points (Fig. 1E). Thus, the higher rates of growth, PC and PN production observed in mid- and high-CO_2_ treatments can be attributed to the elevated *p*CO_2_. Furthermore, the differences between treatments observed in the early stationary growth phase reflect low-end estimates of potential *p*CO_2_ enhancements: cell densities were highest in the elevated CO_2_ treatments, possibly leading to more severe growth limitations and dampening evidence for CO_2_ enhancement.

Our study allows for new insights into the response of *Crocosphaera* to enhanced *p*CO_2_ under a variable *p*CO_2_ environment. Elevated mean *p*CO_2_ enhanced the growth of *Crocosphaera* cultures despite large *p*CO_2_ fluctuations on timescales of less than a generation time, showing that this organism does not need to be acclimated to a stable *p*CO_2_ regime to benefit from elevated *p*CO_2_. Testing the response of microbes to CO_2_ perturbations on multiple timescales is ecologically relevant, because net community metabolism, temperature and salinity effects on CO_2_ solubility, and advective processes cause *p*CO_2_ fluctuations on episodic, diurnal, and seasonal timescales. Phytoplankton will experience the long-term OA *p*CO_2_ signal superimposed onto this existing *p*CO_2_ variability. Furthermore, future OA will increase the DIC:TA ratio of surface waters, leading to a reduced capacity to buffer processes like photosynthesis and respiration, ultimately increasing the magnitude of *p*CO_2_ fluctuations [42].

The *p*CO_2_ fluctuations observed in our study are probably more extreme than the natural variability experienced by *Crocosphaera* populations in open-ocean habitats. In the North Pacific Subtropical Gyre, surface *p*CO_2_ varies by ∼20–50 *µ*atm seasonally [43]; mesoscale features in this region may cause biologically induced *p*CO_2_ swings of ∼150 *µ*atm [44]. However, aggregated cells may experience larger *p*CO_2_ swings; for example, *Crocosphaera nifH* genes have been observed associated with *Trichodesmium* colonies [14] and could thus experience more extreme *p*CO_2_ fluctuations during blooms and subsequent crashes in these concentrated-biomass microhabitats [45]. Regardless of the large magnitude of *p*CO_2_ fluctuations employed in our study, our results suggest that elevated mean *p*CO_2_ impacts the growth response of *Crocosphaera* despite short-term variability.

Overall, our study contributes to the growing literature on the response of marine diazotrophs to elevated *p*CO_2_. We observed that growth, PC and PN production rates of *Crocosphaera* WH8501 were enhanced under elevated and variable *p*CO_2_ in both exponential and early stationary growth phases. It should be noted that a recent study by Garcia et al. [46] found that *Crocosphaera* strains WH0401 and WH0402 appear to be fully saturated under present day *p*CO_2_ conditions (∼400 *µ*atm); thus, elevated *p*CO_2_ seems to have strain-specific effects within *Crocosphaera*. Further research investigating how community composition and environmental conditions regulate the response of marine diazotrophs to elevated *p*CO_2_ will be key to predicting whether global rates of N_2_ fixation will increase under future OA scenarios.

## Supporting Information

Table S1 Time series measurements for cultures of *Crocosphaera watsonii* WH8501 grown under three *p*CO_2_ treatments. Measured pH, calculated *p*CO_2_ (*µ*atm, see Methods) and concentrations of particulate carbon (*µ*mol L^−1^; PC), particulate nitrogen (*µ*mol L^−1^; PN), cells (# mL^−1^), and chlorophyll *a* (Chl *a*; *µ*g L^−1^) are provided for every time point available. Data are mean values from three replicate bottles; standard deviations are presented in parentheses. Dashes indicate no data available.
(DOCX)
