# Supplementary material for: Physiological Response of Crocosphaera watsonii to Enhanced and Fluctuating Carbon Dioxide Conditions
Source: PLoS One. 2014 Oct 24;9(10):e110660. doi: 10.1371/journal.pone.0110660 (PMC4208792; doi:10.1371/journal.pone.0110660)
Supplement: Table S1 — Time series measurements for cultures of Crocosphaera watsonii WH8501 grown under three pCO2 treatments. Measured pH, calculated pCO2 (µatm, see Methods) and concentrations of particulate carbon (µmol L−1; PC), particulate nitrogen (µmol L−1; PN), cells (# mL−1), and chlorophyll a (Chl a; µg L−1) are provided for every time point available. Data are mean values from three replicate bottles; standard deviations are presented in parentheses. Dashes indicate no data available. (DOCX) [file pone.0110660.s001.docx]

**Table S1:** Time series measurements for cultures of *Crocosphaera watsonii* WH8501 grown under three *p*CO_2_ treatments. Measured pH, calculated *p*CO_2_ (*μ*atm, see Methods) and concentrations of particulate carbon (*μ*mol L^-1^; PC), particulate nitrogen (*μ*mol L^-1^; PN), cells (# mL^-1^), and chlorophyll *a* (Chl *a*; *μ*g L^-1^) are provided for every time point available. Data are mean values from three replicate bottles; standard deviations are presented in parentheses. Dashes indicate no data available.

|  | **Low-CO_2_ treatment** | | | | | | **Mid-CO_2_ treatment** | | | | | | **High-CO_2_ treatment** | | | | | |
| --- | --- | --- | --- | --- | --- | --- | --- | --- | --- | --- | --- | --- | --- | --- | --- | --- | --- | --- |
| **Time point** | **pH** | ***p*CO_2_** | **PC** | **PN** | **Cells** | **Chl a** | **pH** | ***p*CO_2_** | **PC** | **PN** | **Cells** | **Chl a** | **pH** | ***p*CO_2_** | **PC** | **PN** | **Cells** | **Chl a** |
| Day 0 L6 | 8.25 (0.01) | 355 (14) | 124 (12) | 8.8 (1) | - | 4.3 (0.6) | 8.07 (0.01) | 600 (21) | 120 (4) | 8.5 (0.7) | - | 4.6 (0.3) | 7.97 (0.01) | 788 (11) | 118 (2) | 7.6 (0.1) | - | 3.9 (0.4) |
|  |  |  |  |  |  |  |  |  |  |  |  |  |  |  |  |  |  |  |
| Day 0 L12 | 8.28 (0.03) | 327 (35) | - | - | - | - | 8.11 (0) | 535 (3) | - | - | - | - | 7.98 (0.01) | 755 (11) | - | - | - | - |
|  |  |  |  |  |  |  |  |  |  |  |  |  |  |  |  |  |  |  |
| Day 1 D12 | 8.23 (0.01) | 376 (10) | - | - | - | - | 8.02 (0.01) | 686 (16) | - | - | - | - | 7.9 (0.02) | 946 (40) | - | - | - | - |
|  |  |  |  |  |  |  |  |  |  |  |  |  |  |  |  |  |  |  |
| Day 1 L6 | 8.32 (0.01) | 288 (8) | 166 (24) | 14.2 (2) | 5.6E+05 (7.4E+04) | 7.3 (1.6) | 8.13 (0.02) | 504 (22) | 192 (1) | 16.2 (0.2) | 8.0E+05 (6.3E+04) | 9 (0.4) | 8.03 (0.02) | 665 (28) | 172 (21) | 13.2 (2) | 7.5E+05 (1.9E+04) | 7.9 (0.4) |
|  |  |  |  |  |  |  |  |  |  |  |  |  |  |  |  |  |  |  |
| Day 1 L12 | 8.38 (0.02) | 237 (14) | - | - | - | - | 8.21 (0) | 405 (6) | - | - | - | - | 8.09 (0.02) | 570 (38) | - | - | - | - |
|  |  |  |  |  |  |  |  |  |  |  |  |  |  |  |  |  |  |  |
| Day 2 D12 | 8.26 (0.02) | 344 (18) | - | - | - | - | 8.02 (0.01) | 693 (19) | - | - | - | - | 7.89 (0) | 963 (4) | - | - | - | - |
|  |  |  |  |  |  |  |  |  |  |  |  |  |  |  |  |  |  |  |
| Day 2 L6 | 8.41 (0.02) | 217 (12) | 374 (145) | 22 (4) | 7.4E+05 (1.2E+05) | 11.6 (1.5) | 8.23 (0.02) | 374 (20) | 424 (73) | 28.2 (3) | 1.2E+06 (1.1E+05) | 16.3 (3) | 8.13 (0.01) | 505 (17) | 547 (63) | 28.3 (2) | 1.2E+06 (9.7E+03) | 15.1 (1) |
|  |  |  |  |  |  |  |  |  |  |  |  |  |  |  |  |  |  |  |
| Day 2 L12 | 8.49 (0.03) | 168 (16) | 555 (88) | 25.3 (3) | 8.7E+05 (1.2E+05) |  | 8.33 (0.02) | 280 (19) | 614 (78) | 27.8 (0.5) | 1.4E+06 (1.1E+05) | - | 8.21 (0.06) | 401 (73) | 521 (80) | 25.4 (3) | 1.5E+06 (1.6E+05) | - |
|  |  |  |  |  |  |  |  |  |  |  |  |  |  |  |  |  |  |  |
| Day 3 D6 | 8.37 (0.02) | 246 (13) | 285 (4) | 30.4 (0.7) | 8.8E+05 (1.1 E+05) | - | 8.11 (0.03) | 531 (50) | 307 (40) | 32.9 (5) | 1.4E+06 (1.2E+05) | - | 7.97 (0.05) | 794 (104) | 300 (33) | 33.3 (4) | 1.4E+06 (4.4E+04) | - |
|  |  |  |  |  |  |  |  |  |  |  |  |  |  |  |  |  |  |  |
| Day 3 D12 | 8.27 (0.01) | 336 (12) | 302 (57) | 39.1 (6) | 9.1E+05 (1.3 E+05) | - | 7.98 (0.01) | 762 (13) | 329 (38) | 52.7 (7) | 1.5E+06 (9.4E+04) | - | 7.85 (0) | 1083 (11) | 312 (35) | 56.2 (5) | 1.5E+06 (7.9E+04) | - |
|  |  |  |  |  |  |  |  |  |  |  |  |  |  |  |  |  |  |  |
| Day 3 L6 | 8.48 (0.05) | 176 (25) | 542 (65) | 42.5 (6) | 1.4E+06 (2.5 E+05) | 24.8 (3) | 8.35 (0.05) | 265 (39) | 706 (88) | 55.4 (6) | 2.4E+06 (1.9E+05) | 39.6 (6) | 8.26 (0.04) | 342 (46) | 740 (44) | 55.9 (4) | 2.6E+06 (1.3E+05) | 35.3 (6) |
|  |  |  |  |  |  |  |  |  |  |  |  |  |  |  |  |  |  |  |
| Day 3 L12 | 8.62 (0.06) | 111 (22) | 821 (85) | 44.2 (6) | 1.5E+06 (1.9E+05) | - | 8.53 (0.05) | 149 (27) | 843 (139) | 49.9 (9) | 2.6E+06 (2.9E+05) | - | 8.41 (0.1) | 226 (75) | 970 (97) | 55.3 (4) | 2.7E+06 (1.4E+05) | - |
|  |  |  |  |  |  |  |  |  |  |  |  |  |  |  |  |  |  |  |
| Day 4 D6 | 8.43 (0.04) | 204 (22) | 494 (63) | 48.1 (6) | 1.6E+06 (2.2E+05) | - | 8.19 (0.03) | 425 (38) | 622 (106) | 69.2 (9) | 2.8E+06 (3.1E+05) | - | 7.99 (0.1) | 768 (213) | 595 (33) | 72.5 (10) | 3.0E+06 (1.7E+05) | - |
|  |  |  |  |  |  |  |  |  |  |  |  |  |  |  |  |  |  |  |
| Day 4 D12 | 8.27 (0.02) | 335 (24) | 465 (88) | 65.6 (7) | - | - | 7.96 (0) | 816 (6) | 670 (91) | 95.3 (6) | - | - | 7.83 (0.02) | 1128 (55) | 718 (43) | 104 (6) | - | - |
|  |  |  |  |  |  |  |  |  |  |  |  |  |  |  |  |  |  |  |
| Day 4 L6 | 8.56 (0.05) | 134 (23) | 833 (121) | 66 (7) | 2.3E+06 (3.1E+05) | 31.6 (2.6) | 8.52 (0.05) | 153 (25) | 1144 (174) | 91.7 (10) | 3.6E+06 (1.5E+06) | 50.6 (9) | 8.47 (0.06) | 180 (33) | 1308 (121) | 97.4 (7) | 4.8E+06 (2.1E+05) | 49.1 (9) |
|  |  |  |  |  |  |  |  |  |  |  |  |  |  |  |  |  |  |  |
| Day 4 L12 | 8.74 (0.08) | 74 (20) | 888 (96) | 64.9 (8) | 2.4E+06 (2.3E+05) | - | 8.73 (0.05) | 74 (13) | 1226 (182) | 88 (12) | 4.6E+06 (5.1E+05) | - | 8.65 (0.12) | 104 (47) | 1351 (118) | 95.4 (10) | 5.1E+06 (2.9E+05) | - |
|  |  |  |  |  |  |  |  |  |  |  |  |  |  |  |  |  |  |  |
| Day 5 D6 | 8.48 (0.07) | 179 (38) | 770 (93) | 72.1 (9) | 2.5E+06 (2.4E+05) | - | 8.24 (0.07) | 369 (74) | 1105 (151) | 109.3 (15) | 5.0E+06 (5.8E+05) | - | 8.03 (0.15) | 694 (303) | 1137 (153) | 124.9 (8) | 5.8E+06 (3.3E+05) | - |
|  |  |  |  |  |  |  |  |  |  |  |  |  |  |  |  |  |  |  |
| Day 5 D12 | 8.25 (0.05) | 355 (51) | 689 (64) | 94.9 (6) | 2.2E+06 (2.4E+05) | - | 7.91 (0) | 925 (8) | 964 (119) | 146.1 (12) | 4.7E+06 (4.5E+05) | - | 7.81 (0.04) | 1216 (127) | 1119 (96) | 166.4 (5) | 5.5E+06 (1.8E+05) | - |
|  |  |  |  |  |  |  |  |  |  |  |  |  |  |  |  |  |  |  |
| Day 5 L6 | 8.61 (0.04) | 115 (15) | 1037 (58) | 84.2 (3) | 3.3E+06 (2.9E+05) | 55.4 (2.1) | 8.59 (0.03) | 119 (14) | 1589 (186) | 130.2 (12) | 6.7E+06 (4.4E+05) | 82.5 (8) | 8.65 (0.02) | 97 (8) | 1852 (65) | 155.1 (2) | 8.1E+06 (3.8E+05) | 101 (2) |
|  |  |  |  |  |  |  |  |  |  |  |  |  |  |  |  |  |  |  |
